# Prognostic Value of Global Longitudinal Strain in Asymptomatic Aortic Stenosis: A Systematic Review and Meta-Analysis

**DOI:** 10.3389/fcvm.2022.778027

**Published:** 2022-02-18

**Authors:** Yuan Wang, Minghui Zhang, Hui Chen, Hongwei Li

**Affiliations:** Affiliated Beijing Friendship Hospital, Capital Medical University, Beijing, China

**Keywords:** impaired global longitudinal strain, asymptomatic aortic stenosis, prognostic value, echo, MACE

## Abstract

**Backgrounds:**

The presence of impaired global longitudinal strain (GLS) may be a valuable bio-marker in the early diagnosis for left ventricle (LV) impairment, which would help scrutinize asymptomatic aortic stenosis (AS) patients with high risk of adverse outcomes, such as major adverse cardiovascular events (MACE).

**Methods:**

The study was prospectively registered in PROPSERO (CRD 42021223472). Databases, such as Pubmed, Embase, Cochrane Library, Web of science, and Scopus were searched for studies evaluating the impact of impaired GLS on MACE, all-cause mortality, and aortic valve replacement (AVR) in asymptomatic AS. Hazard ratios (*HR*s) with 95% *CI*s were calculated with meta-analysis for binary variants. Meta-regression, subgroup analysis, and sensitivity analyses were applied as needed to explore the heterogeneity.

**Results:**

Eventually, a total of nine studies reporting 1,512 patients were enrolled. Compared with the normal GLS group, impaired GLS significantly increased MACE (*HR* = 1.20, 95% *CI*: 1.10–1.30, *I*^2^ = 79%) with evident heterogeneity, all-cause mortality (*HR* = 1.42, 95% *CI*: 1.24–1.63), and AVR (*HR* = 1.17, 95% *CI*: 1.07–1.28). Subgroup analyses stratified by left ventricular ejection fraction (LVEF) > 50% or LVEF without precise cut-off point found that compared with the normal GLS group, impaired GLS remarkably increased MACE both in two subgroups (LVEF > 50%: *HR*: 1.22, 95% *CI*: 1.05–1.50; LVEF without cutpoint: *HR*: 1.25, 95% *CI*: 1.05–1.50). The results stratified by AS severity (mild/moderate and severe) or follow-up time resembled those stratified by LVEF. In addition, when subgroup analysis was stratified by mean aortic valve pressure gradient (MG ≥ 40 mm Hg and MG <40 mm Hg), compared with normal GLS, impaired GLS significantly increased MACE both in two subgroups (MG ≥ 40 mm Hg: *HR*: 3.41, 95% *CI*: 1.64–7.09; MG below 40 mm Hg: *HR*: 3.17, 95% *CI*: 1.87–5.38). Moreover, the effect sizes here were substantially higher than those in the former two stratified factors.

**Conclusions:**

The presence of impaired GLS substantially worsens the outcomes for adverse cardiovascular events in asymptomatic patients with AS regardless of LVEF or AS severity or follow-up time or mean aortic valve pressure gradient, which highlights the importance of incorporating impaired GLS into risk algorithms in asymptomatic AS.

**Systematic Review Registration:**

PROSPERO (registration number: CRD42021223472).

## Introduction

With increased life expectation and lifespan of the population, aortic stenosis (AS) has become one of the most common valvular heart diseases (1). Of note, ~40–50% of severe patients with AS are asymptomatic (2). Although patients with AS can be asymptomatic due to the provisionally sufficient LV function, the myocardial fibrosis resulting from the rising hemodynamic burden could lead to ventricular remodeling and enlargement, which further predisposes patients to sudden cardiac death (3). The progressive exacerbation of left ventricular (LV) dysfunction remains to be a matter of concern in asymptomatic patients.

Currently, left ventricular ejection fraction (LVEF) is used as the main criterion to select asymptomatic patients for aortic valve replacement (AVR) (4). However, there has been growing awareness that LVEF-based hierarchies may have significant deficiencies for early identification of asymptomatic patients with AS who required interventions since the LVEF assessment is highly affected by hemodynamic load. Thus, the LV deterioration might be cloaked in the setting of reduced after-load, where a more sensitive biomarker to facilitate the recognition of LV systolic impairment is urged.

Left ventricular global longitudinal strain (LVGLS), evaluating the contractile function of the ventricular muscle fiber, appears to be a robust parameter for detecting early LV dysfunction, even before the evident deterioration of LVEF (5). Simultaneously, an increasing number of literature published with regard to LVGLS in asymptomatic patients with AS and its association with adverse outcomes. However, most studies were from the single-center with relatively small sample size.

Given the increasing sound of LVGLS in evaluating asymptomatic patients with AS, we sought to perform a systematic review and meta-analysis to evaluate the prognostic value of LVGLS among asymptomatic patients with AS.

## Methods

### Study Search

The present study was conducted in accordance with the Preferred Reporting Items for Systematic Reviews and Meta-Analysis statement (PRISMA-NMA) (6). The original study protocol was registered prospectively in PROSPERO (registration number: CRD42021223472).

A comprehensive systematic search was performed by two independent investigators (YW and MHZ) in electronic databases (Pubmed, Embase, Cochrane Library, Web of Science, and Scopus) for the period up to June 16, 2021. The detailed mesh terms were: AS, GLS, asymptomatic, echocardiography, speckle tracking, progression, major adverse cardiovascular events (MACE), all-cause mortality, and AVR. The full search strategy is provided in the Supplementary Table 1. To ensure saturation, a manual reference cross-check of included articles was performed.

### Inclusion Criteria

1) Left ventricular global longitudinal strain was evaluated by speckle tracking in patients with asymptomatic AS;
2) Binary clinical outcome data (e.g., all-cause mortality) with follow-up time of at least 3 months were reported in patients stratified by the presence of abnormal LVGLS;
3) Sufficient data to retrieve hazard ratios (HRs) and corresponding 95% *CI*s.

### Exclusion Criteria

1) Studies assessing basal longitudinal strain (BLS) instead of GLS or lack of our prespecified outcomes;
2) Studies not based on humans;
3) Case reports, comments, reviews, abstracts, meta-analyses, and editorials;
4) Duplicate publications.

### Data Extraction and Quality Assessment

Two investigators (YW and MHZ) independently evaluated studies for eligibility and extracted data from enrolled studies. When the study comprised both the symptomatic and asymptomatic patients with AS, only the data of asymptomatic AS were collected. The following information were collected: the first name of author, year of publication, sample size, study design, follow-up duration, patient baseline characteristics, echocardiographic parameters (such as, imaging modalities for quantifications of GLS), the withdrawal rate, outcomes, etc. Any discrepancy was adjudicated by a senior investigator (HWL).

The Newcastle-Ottawa Quality Assessment Scale was implemented to assess the quality of enrolled studies (Supplementary Table 2) (7). The quality of the selected trials was determined on the basis of selection of the study groups (0–4 points), comparability of the study groups (0–2 points), and ascertainment of the outcome of interest (0–3 points). The studies scored ≥7 were considered to be high quality articles.

### Endpoints

The primary endpoint was MACE. MACE comprises cardiac death, AVR, and any cardiovascular events (hospital admission for heart failure, acute myocardial infarction, ventricular tachyarrhythmia, or stroke). The secondary endpoint was all-cause mortality. The tertiary endpoint was AVR.

### Statistical Analysis

Hazard ratios with 95% *CI*s were calculated with meta-analysis for binary variants. If the trials did not report *HR*s directly, Kaplan–Meier curves were read using Engauge Digitizer version 4.1 (8). If *I*^2^ <50% and *p* > 0.01, a fixed effect model would be adopted, otherwise a random-effect model would be performed. If there was obvious heterogeneity, meta-regression, subgroup analysis and sensitivity analyses would be applied. The *p* < 0.05 was considered statistically significant. Publication bias was tested using funnel plots, the Egger's test, and the Peter's test (9, 10). The Duval and Tweedie's trim and fill method was employed to further assess the possible effect of publication bias (11). All statistical analyses were performed by using R version 3.5.3 (R Foundation for Statistical Computing, Vienna, Austria). A two-tailed *p* < 0.05 was considered statistically significant.

## Results

### Study Selection

**Figure 1:**
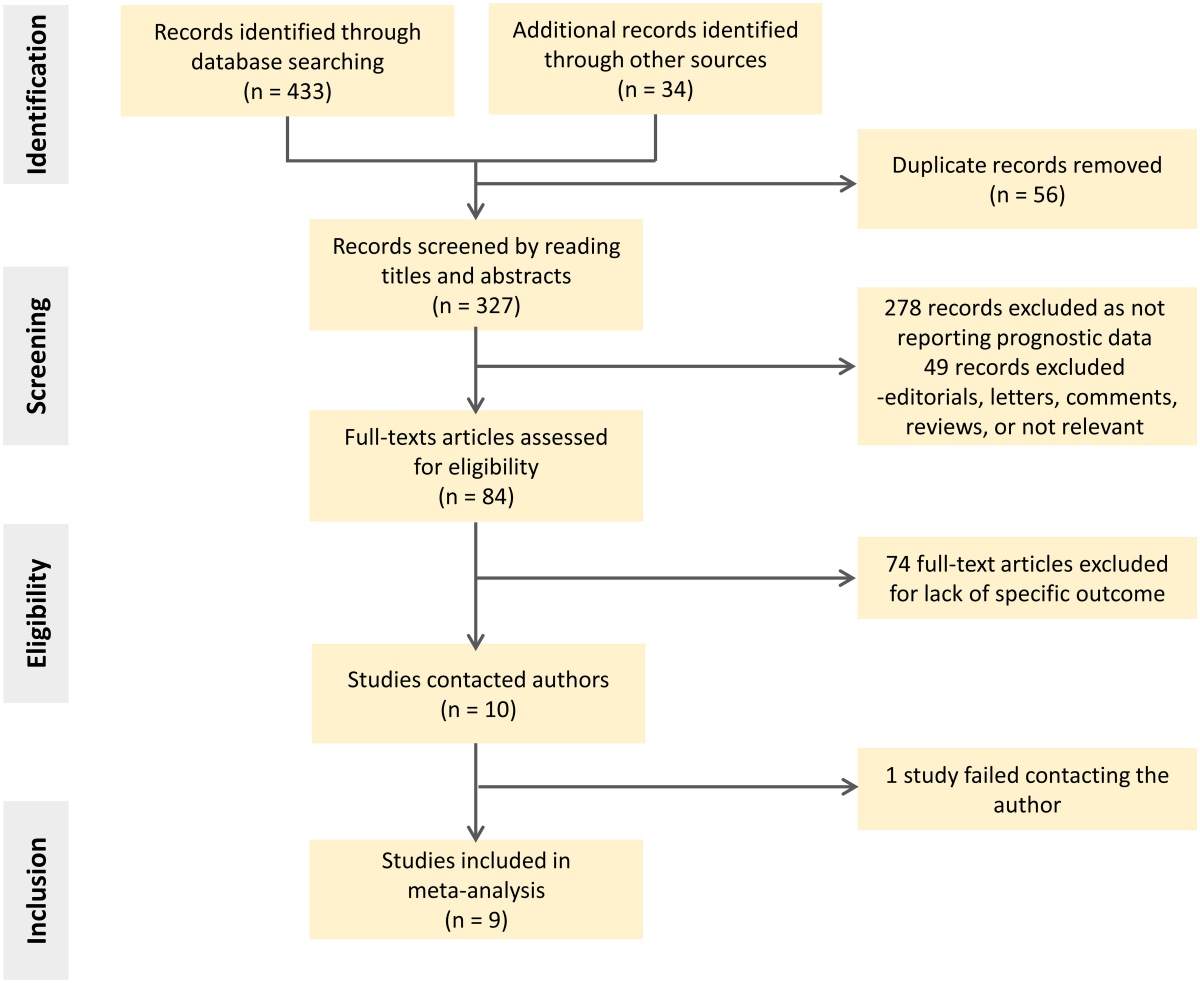
Study search and selection flowchart.

The search identified 467 citations based on title/abstract, from which 327 full-text reports were carefully assessed. Of these, 318 studies were excluded for various reasons, such as assessing basal longitudinal strain (BLS) instead of GLS or lack of our prespecified outcomes. The screening flowgram is presented in Figure 1.

### Characteristics of Selected Studies

Eventually, a total of nine studies with 1,512 participants were included (12–20). Table 1 shows the summary of included studies. All studies were published from 2010 to 2021, of which three were multicenter. Five of them were based on prospective cohorts. The sample size varied form *n* = 52 patients to *n* = 332 patients. The incidence of impaired-GLS ranged from 37.5 to 62.7% over a follow-up duration ranging from 11 to 33.4 months. The cutoff value of GLS associated with MACE was derived from a receiver operating characteristic (ROC) curve analysis in five studies (14–16, 19, 20). Two of the rest used the median value of its sample (12, 17). Only two studies chose the clinical cut point (13, 18). Additional information regarding the study endpoint can be found in Supplementary Table 3. The bias risk of the enrolled studies was found to be low based on the Newcastle-Ottawa Quality Assessment Scale (Supplementary Table 2).

**Table 1:** Description of studies included in meta-analysis.

**References**	**Type of study**	***n***	**Population considered for meta-analysis**	**Abnormal GLS-positive**	**Cut-off of GLS**	**Mean or median of follow-up**	**Withdrawal rate**
Kitano et al. (12)	Retrospective, monocentric, consecutive pts	325^*^	asy-AS	162 (49.8%)	15.1	24 months	4.41%
Thellier et al. (13)	Retrospective, monocentric, consecutive pts	332	severe asy-AS, EF ≥ 50%	192 (57.8%)	15	42 months	0%
Gu et al. (14)	Retrospective, monocentric, consecutive pts	218	moderate to severe asy-AS, EF ≥ 50%	131 (60.1%)	15	33.4 months	0%
Carstensen et al. (15)	Prospective, multicentric	104	moderate to severe asy-AS, EF ≥ 50%	39 (37.5%)	15	2.3 yrs	0%
Nagata et al. (16)	Retrospective, multicentric	104	severe asy-AS, EF > 50%	64 (62.7%)	17	373 days	1.92%
Yingchoncharogen et al. (17)	Prospective, monocentric	79	severe asy-AS, EF ≥ 50%	NA	NA	16 months	0%
Kearney et al. (18)	Prospective, monocentric, consecutive pts	135^*^	mild to moderate asy-AS	54 (40.0%)	15	2.1 yrs	7.53%
Zito et al. (19)	Prospective, monocentric	52	severe asy-AS, EF ≥ 50%	NA	NA	11 months	16.92%
Lancellotti et al. (20)	Prospective, multicentric	163	moderate to severe asy-AS, EF ≥ 55%	79 (48.5%)	15.9	20 months	0%

*Values are expressed as n (%). Asy-AS, asymptomatic aortic valve stenosis; EF, ejection fraction; GLS, global longitudinal strain; NA, not applicable*.

* *The population considered for this meta-analysis is a part of the total population of the original study*.

Patients were predominantly men (67%), with a mean age of 75 years (Table 2). These studies covered a wide spectrum of patients with AS, with a mean pressure gradient ranging from 24 to 60 mm Hg (Table 3). Follow-up periods were mostly more than 12 months. The majority of studies used GE echocardiographic platforms and Echopac to analyze the strain data. Of note, two studies (Thellier et al. and Nagata et al.) performed with both GE and Philips equipment used Tomtec imaging system for the strain analysis (13, 16).

**Table 2:** Clinical characteristics of patients.

**References**	**Male**	**Age (yrs)**	**BMI (kg/m^**2**^)**	**BSA (m^**2**^)**	**Hypertension**	**Diabetes**	**Dyslipidemia**	**CAD**	**CKD**
Kitano et al. (12)	152 (45%)	77 ± 10	22.7 ± 3.9	1.52 ± 0.21	273 (80%)	111 (33%)	126 (37%)	74 (22%)	161 (47%)
Thellier et al. (13)	136 (41%)	79 (71–85)	27.1 (23.9–31.3)	1.84 (1.70–1.98)	235 (71%)	118 (36%)	NA	77 (23%)	NA
Gu et al. (14)	117 (54%)	69 ± 14	28.0 ± 4.9	NA	172 (79%)	42 (19%)	NA	86 (39%)	52 (24%)
Carstensen et al. (15)	71 (68%)	72 ± 9	26.8 ± 4.0	NA	71 (68%)	13 (13%)	61 (59%)	15 (14%)	NA
Nagata et al. (16)	43 (41%)	78 ± 10	22.5 ± 2.7	1.50 ± 0.17	67 (64%)	21 (20%)	42 (40%)	19 (18%)	48 (46%)
Yingchoncharogen et al. (17)	39 (49%)	77 ± 12	29 ± 5	NA	50 (63%)	19 (24%)	57 (72%)	NA	NA
Kearney et al. (18)	91 (67%)	75 ± 11	NA	NA	116 (79%)	38 (26%)	NA	58 (40%)	NA
Zito et al. (19)	17 (33%)	72 ± 11	NA	NA	15 (29%)	4 (8%)	13 (25%)	NA	NA
Lancellotti et al. (20)	106 (65%)	70 ± 10	NA	NA	81 (50%)	27 (17%)	72 (44%)	NA	NA

*Values are expressed as mean ± SD or n (%)*.

*BSA, body surface area; BMI, body mass index; CAD, coronary artery disease; CKD, chronic kidney disease; NA, not applicable*.

**Table 3:** Echocardiographic parameters of the included studies.

**References**	**AVA (cm^**2**^)**	**iAVA (cm^**2**^/m^**2**^)**	**Mean PG (mmHg)**	**AV Vmax (m/s)**	**LVEF (%)**	**LV-GLS (%)**	**SVI (ml/m^**2**^)**	**LVMI (g/m^**2**^)**	**E/A**	**E/e'**	**Platform (Vendor)**	**Analysis software**	**Reproducibility of GLS performed**
Kitano et al. (12)	1.05 ± 0.36	0.70 ± 0.25	24 ± 14	3.1 ± 0.8	50.7 ± 6.5	15.0 ± 3.6	45 ± 12	116 ± 35	0.85 ± 0.48	19.4 ± 9.8	Philips (IE 33 or Epic 7G)	Philips Medical Systems	NA
Thellier et al. (13)	0.85 (0.70–1.00)	0.47 (0.39–0.55)	35 (24–45)	3.80 (3.14–4.29)	61 (57–66)	14.1 (11.0–16.7)	39 (32–44)	108 (90–129)	NA	NA	GE (Vivid E9, Vivid E95, Vivid 7), Philips (IE 33 or Epiq 7)	Tomtec Imaging Systems	Y
Gu et al. (14)	0.96 ± 0.30	0.51 ± 0.16	32 ± 12	NA	65.3 ± 8.2	16.3 ± 4.5	32.6 ± 10.3	103 ± 35	NA	11.5 ± 5.3	GE (Vivid 7)	EchoPac	Y
Carstensen et al. (15)	0.90 (0.75–1.14)	0.47 (0.39–0.60)	24 (18–40)	3.3 (2.8–4.0)	61 (57–66)	15.6 ± 2.7	42 (36–49)	83 (72–98)	0.89 (0.77–1.13)	NA	GE (Vivid E9)	EchoPac	Y
Nagata et al. (16)	NA	0.42 ± 0.10	39.4 ± 17.1	4.05 ± 0.80	60 ± 5	15.8 ± 3.4	38 ± 10	89 ± 23	0.77 ± 0.38	19.5 ± 8.4	GE (Vivid E9, Vivid 7), Philips (IE 33)	Tomtec Imaging Systems	NA
Yingchoncharogen et al. (17)	0.75 ± 0.12	NA	36.8 ± 12.6	4.4 ± 0.3	63.4 ± 7.9	15.16 ± 2.49	NA	101.2 ± 29.3	0.93 ± 0.51	NA	Siemens	Syngo Velocity Vector Imaging	Y
Kearney et al. (18)	1.0 ± 0.4	NA	40 ± 20	NA	59 ± 11	15 ± 4	NA	120 ± 38	NA	NA	GE (Vivid 7)	EchoPac	Y
Zito et al. (19)	0.6 ± 0.2	0.36 ± 0.11	60 ± 16	NA	61 ± 5	15 ± 4	35 ± 10	127 ± 38	0.8 ± 0.2	18 ± 13	GE (Vivid 7)	EchoPac	NA
Lancellotti et al. (20)	NA	0.45 ± 0.09	46 ± 14	4.2 ± 0.6	66 ± 9	15.7 ± 3.1	NA	91 ± 45	0.99 ± 0.54	NA	GE (Vivid 7)	EchoPac	NA

*Values are mean ± SD, median value (interquartile range) or n (%) unless otherwise indicated*.

*AVA, aortic valve area; AV Vmax, Peak velocity of the aortic valve; GE, General Electric; iAVA, indexed aortic valve area; LVEF, left ventricle ejection fraction; LV-GLS, left ventricular global longitudinal strain; LVMI, left ventricular mass index; PG, pressure gradient; SVI, stroke volume index; Y, yes; NA, not applicable*.

### Overall Analysis

**Figure 2:**
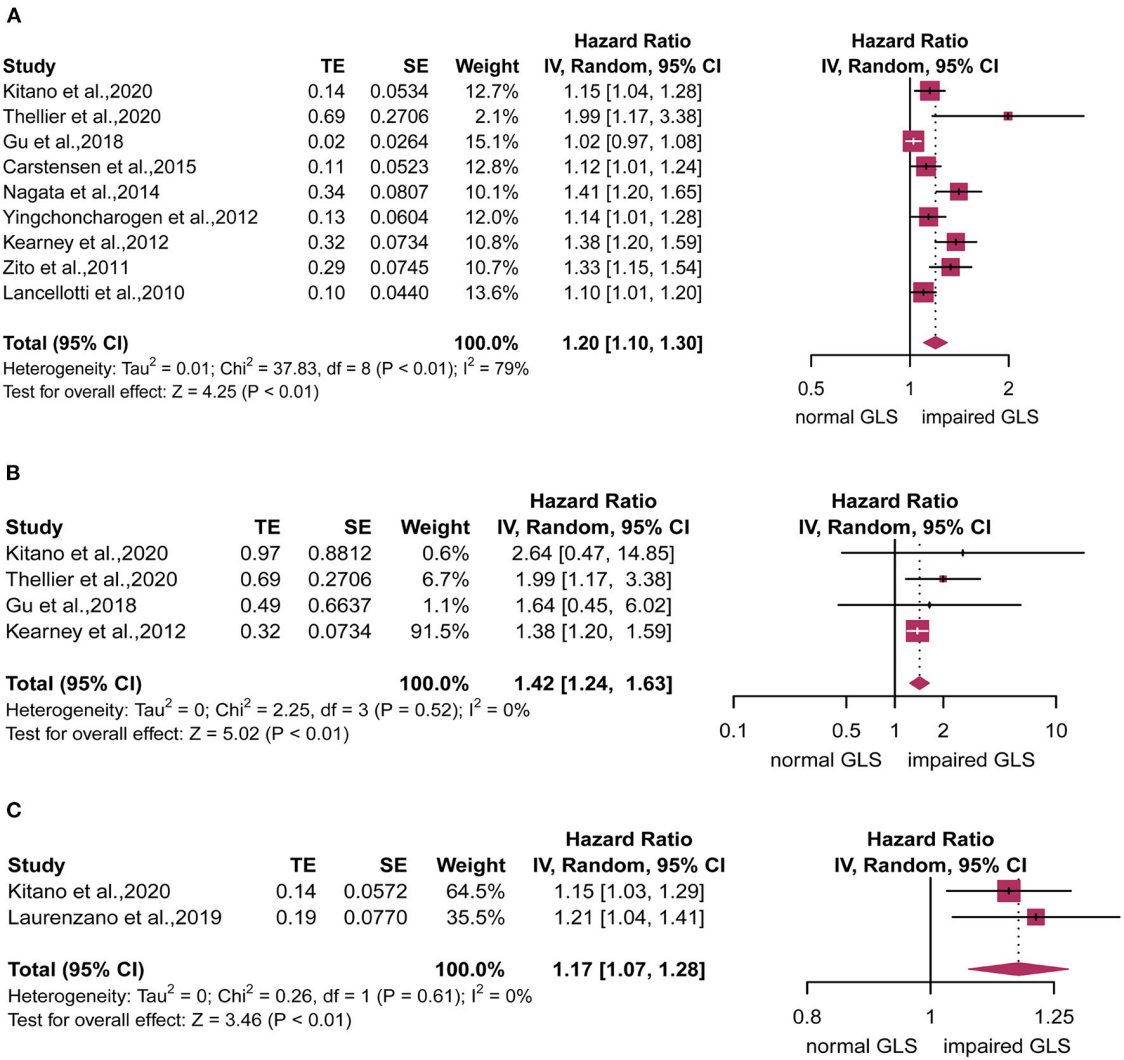
Forest plot for the association between impaired global longitudinal strain (GLS) and **(A)** major adverse cardiovascular events (MACE), **(B)** all-cause mortality, or **(C)** aortic valve replacement (AVR).

For analyzing the primary endpoint, nine of 9 studies reporting 1,512 patients were eligible. Compared with the normal GLS group, impaired GLS significantly increased risk of MACE (*HR*: 1.20, 95% *CI*: 1.10–1.30; *I*^2^ = 79%, *p* < 0.01) with evident heterogeneity (Figure 2A). Four of 9 studies reporting 1,010 patients were eligible for analyzing the secondary endpoint. The pooled estimates showed an increased risk of all-cause mortality in patients with impaired GLS as compared with normal GLS patient (*HR*: 1.42, 95% *CI*: 1.24–1.63) with no statistically heterogeneity (*I*^2^ = 0%, *p* < 0.01) (Figure 2B). Two of 9 studies reporting 488 patients were eligible for analyzing the tertiary endpoint. Compared with normal GLS group, impaired GLS significantly increased incidence of AVR (*HR*: 1.17, 95% *CI*: 1.07–1.28; *I*^2^ = 0%, *p* < 0.01) (Figure 2C).

### Sensitivity Analysis and Meta-Regression Analysis

We performed the leave-one-out sensitivity analysis. Of note, the individual exclusion of studies did not alter the merged effect sizes (Supplementary Figure 1). Meta-regression was performed stratified, respectively, by age, gender, comorbidities (hypertension, diabetes mellitus, and coronary artery disease), and echocardiographic data (baseline EF, baseline GLS, GLS cutoff values, and mean pressure gradient) but failed to find any correlation (Supplementary Table 4).

### Subgroup Analysis

**Figure 3:**
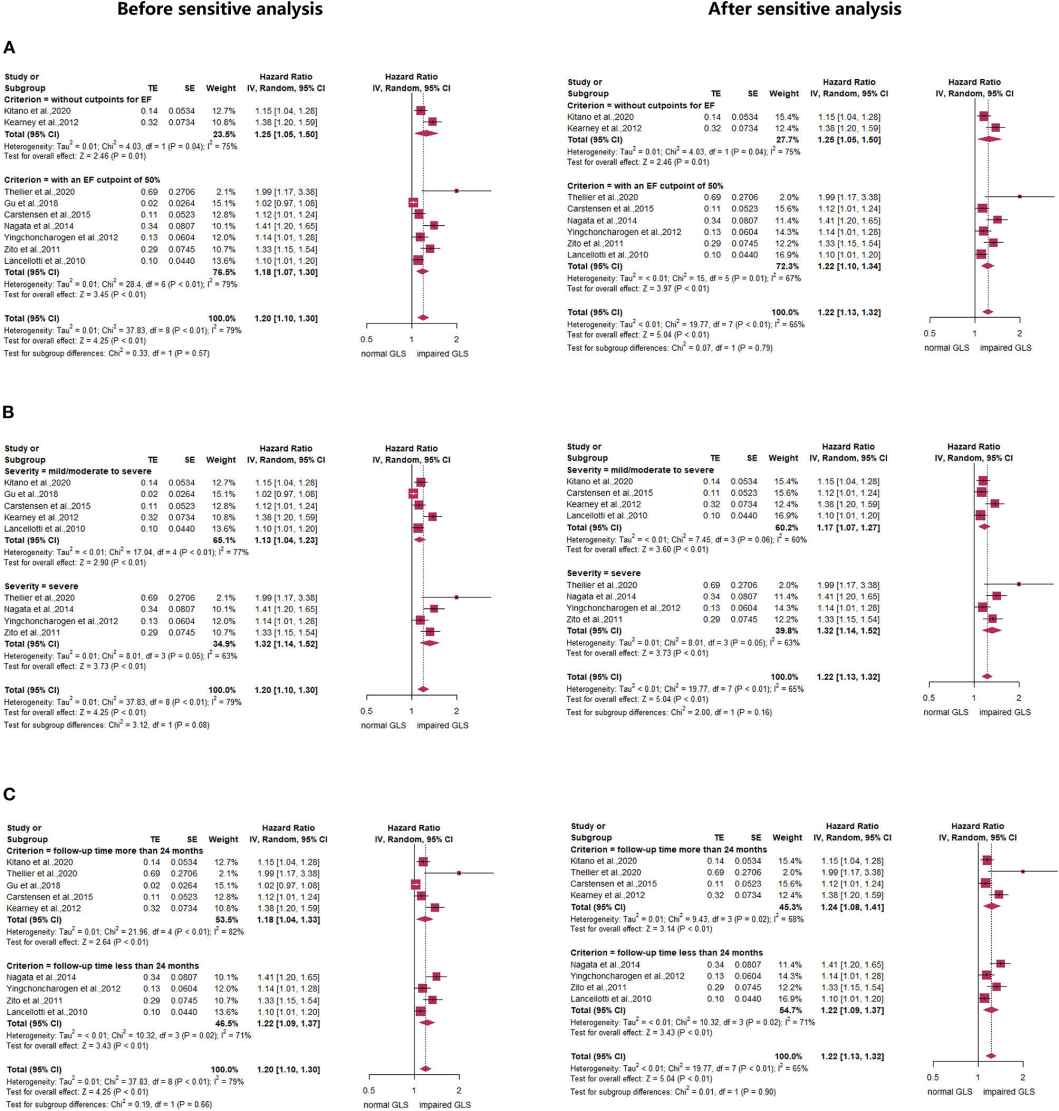
Forest plot demonstrating the association between impaired GLS and MACE defined by **(A)** ejection fraction (EF) or **(B)** aortic stenosis (AS) severity or **(C)** follow-up time.

**Figure 4:**
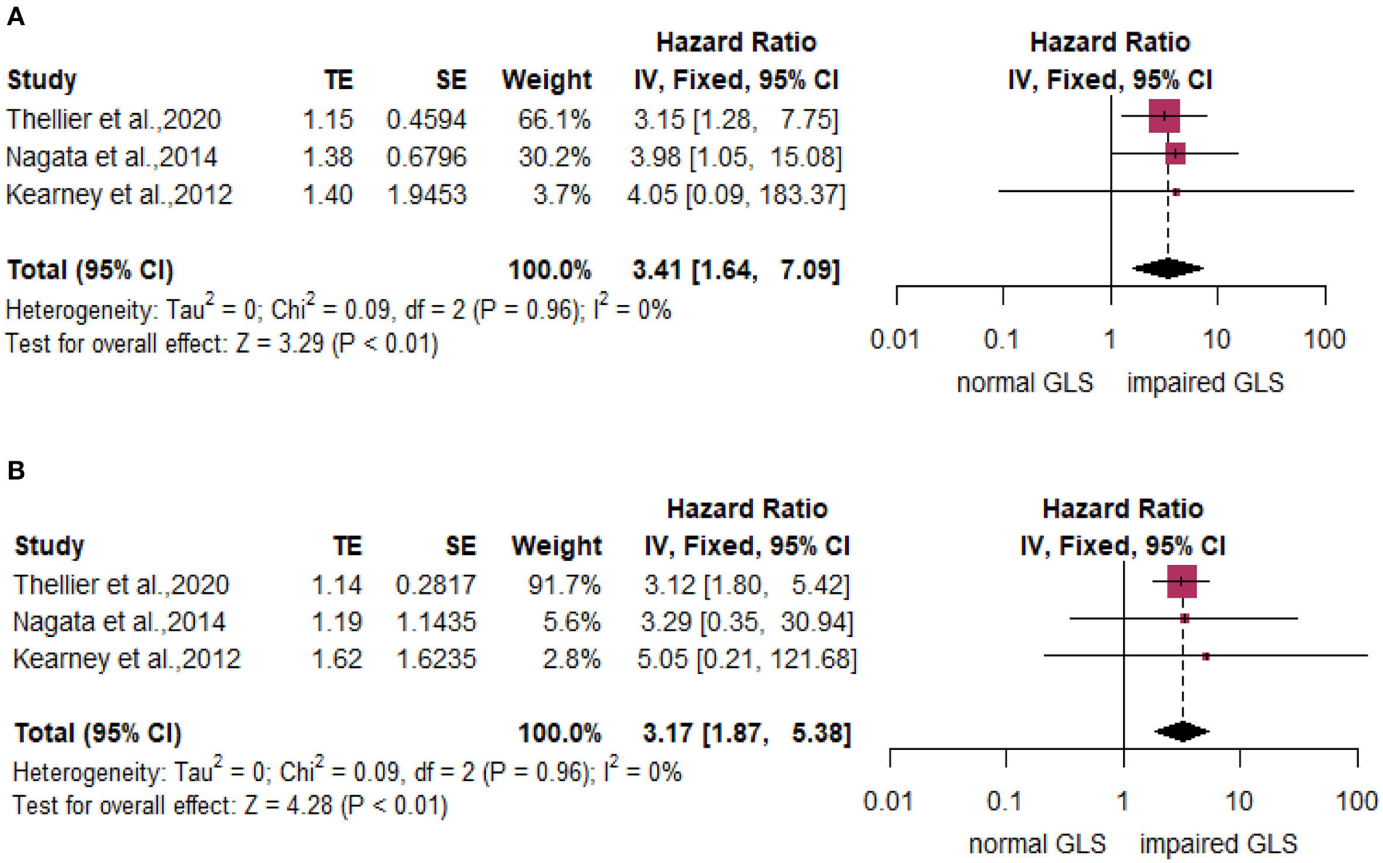
Forest plot demonstrating the association between impaired GLS and MACE dichotomized by mean aortic valve pressure gradient (MG) over or below 40 mm Hg. **(A)** MG ≥ 40 mmHg. **(B)** MG <40 mmHg.

Subgroup analyses stratified by LVEF of 50% found that compared with the normal GLS group, impaired GLS remarkably increased MACE both in two subgroups (LVEF > 50%: *HR*: 1.22, 95% *CI*: 1.05–1.50; LVEF without cut-off point: *HR*: 1.25, 95% *CI*: 1.05–1.50). There was no statistically significant difference between these two subgroups (*p* = 0.79) (Figure 3A, right panel). In addition, the heterogeneity decreased from 79 to 65% after excluding Gu's study, which indicated that inclusion of the study by Gu et al., is among the main cause of heterogeneity. The results stratified by AS severity (mild/moderate and severe) (mild/moderate AS: *HR* = 1.17, 95% *CI*: 1.07–1.27; severe AS: *HR*: 1.32, 95% *CI*: 1.14–1.52) resembled those stratified by LVEF. The value of *p* for the difference between these two subgroups was not significant at 0.16 (Figure 3B, right panel). The heterogeneity of the subgroup with mild/moderate AS decreased from 77 to 60% after excluding Gu's study. We again found similar results in subgroups stratified by follow-up time (follow-up time more than 24 months: *HR* = 1.24, 95% *CI*: 1.08–1.41; follow-up time less than 24 months: *HR*: 1.22, 95% *CI*: 1.09–1.37; *p* for subgroup difference = 0.90) (Figure 3C, right panel). The heterogeneity of the subgroup with follow-up time more than 24 months decreased from 82 to 68% after excluding Gu's study. The subgroup analysis stratified by mean aortic valve pressure gradient (MG ≥ 40 mm Hg and mean MG <40 mm Hg) found that compared with the normal GLS, impaired GLS significantly increased MACE both in two subgroups (mean MG ≥ 40 mm Hg: *HR*: 3.41, 95% *CI*: 1.64–7.09; MG below 40 mm Hg: *HR*: 3.17, 95% *CI*: 1.87–5.38) (Figure 4). Moreover, the effect sizes here were substantially higher than those in the former two stratified factors.

### Publication Bias

The funnel plot for MACE visually showed mildly asymmetric (Supplementary Figure 2). The public bias was also confirmed by Egger's test (*p* < 0.01) as well as Begg's test (*p* = 0.01). To mitigate against publication bias, we used the trim and fill method. The trim and fill analysis suggested two missing studies. After the imputation of these missing studies, the recalculated pooled *HR* for impaired GLS being associated with MACE of asymptomatic AS was slightly attenuated but remained significant (*HR* 1.16, 95% *CI*: 1.07–1.26).

## Discussion

The present meta-analysis of data from nine studies, including 1,512 patients, identified robust associations between impaired GLS and MACE, all-cause mortality, or AVR across a wide spectrum of patients with asymptomatic AS. Although the heterogeneity in the primitive analysis was high, the subgroup analysis and meta-regression in the current meta-analysis confirms that impaired GLS was a powerful risk marker for MACE with eliminated heterogeneity. Given these findings, impaired GLS had strong prognostic utility in asymptomatic AS populations.

The best timing of AVR for asymptomatic patients with AS has been the epicenter of considerable interest during the last decades (21). According to the latest American and European guidelines, the EF of 50% was used as the threshold for surgery among asymptomatic severe patients with AS (22, 23). However, data from recent published studies demonstrated that singular LVEF might be insufficient to risk-stratify those patients for surgery (24, 25). LVEF values could be falsely enhanced by the geometric effect, which might limit its ability to identify mild or moderate LV dysfunction. Of note, Ng. et al. (26) stated that up to 23 (13.1%) deaths occurred in patients without severely impaired EF, because they fell outside the recommendations. Although the Society of Thoracic Surgeons (STS) introduced exercise-induced symptom as the supplement of criterion, it was latterly found to be subjective and unconvincing (27, 28).

It was well-known that reduced value of deformation and speed longitudinal contraction was prior to LVEF impairment in AS. Thus, GLS has emerged as an accurate and reproducible tool to identify the subclinical LV impairment of AS (5). According to a series of recent studies, identification of reduced longitudinal strain rate caused by the injured subendocardial myocardial fibers had correlations with mortality among asymptomatic patients with AS (12, 16, 19). However, the prognostic value of GLS in asymptomatic patients with AS has been demonstrated merely in small single-center studies without further confirmation in larger patient populations (12–20).

Only one meta-analysis evaluated the effect of impaired GLS on prognosis in asymptomatic AS patients (29). This meta-analysis, which used individual participant data from 10 studies, demonstrated that patients with impaired GLS, which defined by the cut-off value of 14.7%, had 2-fold greater risk than those with normal GLS over the course of the follow-up. However, it was important to note that this study set mortality as the solo endpoint. In the real life, what we really focus on is not only the mortality, but also the possibility of symptoms appearance and the quality of life, especially for young patients with AS (e.g., patients with bicuspid aortic valve). Hence, in compliance of the real-world practice, we chose MACE to be the primary endpoint, by GLS we could predict or screen patients with AS who were likely to progress into symptomatic and required surgery. Furthermore, the prior meta-analysis was limited to several other ways, such as small sample sizes (65–163 patients), the use of BLS instead of GLS in the included study by Carstensen et al. (15), and the inclusion of symptomatic patients with AS against inclusion criteria (30–33). Hence, caution is needed in interpreting the outcomes of this meta-analysis.

Given the wealth of published studies over the past 3 years and the absence of a meta-analysis which specifically focused on the combined endpoint in asymptomatic patients with AS, it is necessary to conduct our analysis. In our study, which addressed several outcomes in a larger patient population compared with the previous meta-analysis, impaired GLS was present in a considerable proportion of patients (52.2%), and it had significant associations with MACE, all-cause mortality, or AVR. The heterogeneity substantial decreased after excluding Gu's study in subgroups stratified by LVEF, AS severity, or follow-up time. The heterogeneity might ascribe to the inaccuracy of GLS in prediction of adverse events in Gu's study. It should be noticed that stronger correlations between impaired GLS and MACE was observed when involving patients with EF > 50% only. Our data implicate that the combination of EF below 50% and impaired GLS might help identify asymptomatic patients with AS at high risk of poor outcomes. One may speculate if early intervention or frequent monitoring might benefit patients with impaired GLS, independent of LVEF.

There was evidence indicative of publication bias. Therefore, we used the trim and fill method to adjust for the publication bias. The trim and fill analysis showed the similarly significant adjusted pooled *HR* for MACE in asymptomatic patients with AS, which indicated that the publication bias had little effect on our results. As research on the topic of GLS in asymptomatic AS is relatively new, manuscripts that report significant results are likely to be of interest to peer-reviewed journals. The unwritten and unpublished null findings might lead to the publication bias. The publication bias might also result from other factors, such as sample size, GLS analysis software, and other complications.

A few studies identified additional prognostic markers, e.g., natriuretic peptide, exercise tolerance, aortic valve calcium score, multidetector row CT (MDCT)-derived GLS, cardiac magnetic resonance (CMR)-derived GLS, extracellular volume assessed by CMR (20, 34–38). Noticeably, GLS is superior to natriuretic peptide, exercise tolerance, aortic valve calcium score in detecting outcomes in AS (20, 39). The study by Kim et al. (40) showed that CMR-derived GLS might be associated with cardiac dysfunction in asymptomatic patients with AS. The extracellular volume or MDCT-derived GLS may add prognostic value beyond what is obtained from GLS (36, 38). However, some methodological limitations of these prognosticators should be addressed. Both MDCT and CMR acquisition are not routinely used among patients with AS. Extracellular volume and CMR-derived GLS require specialized sequences and lengthy breath-holds, which is more time-consuming than echocardiography. In terms of accuracy, sensitivity, and reproducibility, GLS by echo seems to perform better than other prognostic factors. But for patients with suboptimal imaging quality on echo, CMR or MDCT could represent a valid alternative.

Our meta-analysis may have considerable clinical implications. First, the presence of GLS offers opportunities to identify asymptomatic patients with AS who are at high risk of adverse prognosis and therefore act accordingly. This could help reduce costs associated with repeat admissions of normal patients with GLS. Our results may help address the unsolved issue of whether signs of LV impairment could be used to optimize the timing of valve intervention (surgery or TAVI). An ongoing trial (Danish National Randomized Study on Early Aortic Valve Replacement in Patients with Asymptomatic Severe Aortic Stenosis (DANAVR); NCT03972644), which randomly assigns asymptomatic patients with AS to undergo AVR or watchful waiting, would shed more light on this hot issue. Second, for bicuspid aortic valve (BAV) individuals, valve dysfunctions usually occur early in life. Thus, the overall quality of life and ability to re-integrate into society are as important as survival outcomes for these patients. Timely, appropriate management enabled by the early detection of LV impairment is of utmost importance, especially for Asian patients with AS with considerably high proportions of BAVs.

## Limitation

First, all the studies enrolled were observational studies rather than randomized clinical trials, indicating potential confounding factors that might result in bias eventually. However, it must be underscored that no randomized trials have yet been performed on this theme. Second, the sample size of some enrolled studies were small, which might compose part of the bias. Third, as individual patient data were not available, several important covariates, such as medication could not be fully assessed. Nevertheless, the results of our meta-analysis do summarize the outcomes of nine studies in 1,512 individuals, with significant associations between impaired GLS and MACE remained even after adjustment for confounding with subgroup assessment and meta-regression analysis. Fourth, our results might be influenced by the publication bias. But the publication bias may not be totally ruled out due to possibly unpublished studies with non-significant results. However, the trim and fill analyses showed similarly significant results.

## Conclusion

The presence of impaired GLS substantially worsens the outcomes for adverse cardiovascular events in asymptomatic patients with AS regardless of LVEF or AS severity or mean aortic valve pressure gradient, which highlights the importance of incorporating impaired GLS into risk algorithms in asymptomatic AS. Certainly, large scale RCTs are needed to justify our speculation.

## Data Availability Statement

The raw data supporting the conclusions of this article will be made available by the authors, without undue reservation.

## Author Contributions

YW and MZ performed all statistical analyses and echocardiographic analyses. YW contributed to the drafting of the work and design of the study. YW and HC was responsible for the conception of the project. All authors participated in critical revision of the work, and all authors read and approved the final version to be submitted for publication.

## Funding

This study was supported by the Research Grant of National Natural Science Foundation of China (82000361).

## Conflict of Interest

The authors declare that the research was conducted in the absence of any commercial or financial relationships that could be construed as a potential conflict of interest.

## Publisher's Note

All claims expressed in this article are solely those of the authors and do not necessarily represent those of their affiliated organizations, or those of the publisher, the editors and the reviewers. Any product that may be evaluated in this article, or claim that may be made by its manufacturer, is not guaranteed or endorsed by the publisher.

## Supplementary Material

Supplementary Table 1 The full search strategy.

Supplementary Table 2 Quality assessment using Newcastle Ottawa scale.

Supplementary Table 3 The outcomes of included studies.

Supplementary Table 4 Meta-regression of baseline feature on major adverse cardiovascular events (MACE) at follow-up.

Supplementary Figure 1 Sensitivity analyses using the leave-one-out approach.

Supplementary Figure 2 Funnel plot for the association between global longitudinal strain (GLS) and major adverse cardiovascular events (MACE) in asymptomatic patients with aortic stenosis (AS).
