# Supplementary material for: Prognostic Value of Global Longitudinal Strain in Asymptomatic Aortic Stenosis: A Systematic Review and Meta-Analysis
Source: Front Cardiovasc Med. 2022 Feb 18;9:778027. doi: 10.3389/fcvm.2022.778027 (PMC8894446; doi:10.3389/fcvm.2022.778027)
Supplement: Supplementary Table 1 — The full search strategy. [file Table_1.docx]

| **Electronic Databases** | **Full Search String** |
| --- | --- |
| PubMed  (n=89) | (((((“asymptomatic” [All Fields]) OR (“subclinical”[All Fields]) OR ("preserved ejection fraction" [All Fields]) OR ("preserved" [All Fields] AND "ejection fraction" [All Fields])) AND ("aortic valve stenosis" [MeSH Terms] OR ("aortic" [All Fields] AND "valve" [All Fields] AND "stenosis" [All Fields]) OR "aortic valve stenosis" [All Fields] OR "aortic stenosis" [MeSH Terms] OR ("aortic" [All Fields] AND "stenosis" [All Fields]) OR "aortic stenosis" [All Fields] OR "AS" [All Fields])) AND (("global longitudinal strain" [MeSH Terms] OR "global longitudinal strain"[All Fields] OR "GLS" [All Fields] OR "longitudinal strain" [All Fields] OR "strain" [All Fields] OR "speckle tracking" [All Fields]) AND ("echocardiography" [MeSH Terms] OR "echocardiography"[All Fields] OR "echocardiogr*" [All Fields] OR "ultrasound" [All Fields]))) NOT ("case reports" [Publication Type] OR "comment" [Publication Type] OR "editorial" [Publication Type])) |
| Embase  （n=104） | ('asymptomatic' OR 'subclinical' OR 'preserved ejection fraction'/exp OR 'preserved ejection fraction') AND ('aortic valve stenosis'/exp OR 'aortic valve stenosis' OR 'aortic stenosis'/exp OR 'aortic stenosis') AND ('echocardiography'/exp OR 'echocardiography' OR echocardiogr* OR 'ultrasound'/exp OR 'ultrasound') AND ('global longitudinal strain'/exp OR 'global longitudinal strain' OR 'speckle tracking'/exp OR 'speckle tracking' OR 'strain'/exp OR 'strain') AND ([article]/lim OR [article in press]/lim) AND [english]/lim AND [1980-2021]/py |
| Cochrane Library  （n=106） | asymptomatic OR subclinical OR preserved ejection fraction OR (preserved AND ejection fraction) in All Text AND aortic valve stenosis OR aortic stenosis OR AS OR (aortic AND valve) in All Text AND echo* OR ultrasound in All Text AND global longitudinal strain OR speckle tracking OR longitudinal strain OR GLS OR strain in All Text - with Publication Year from 1985 to 2021, in Trials (Word variations have been searched) |
| Web of Science  （n=65） | (TS=[(((((aortic stenosis OR aortic valve stenosis) AND asymptomatic) AND (global longitudinal strain OR longitudinal strain OR speckle tracking)) AND (echocardiography OR ultrasound)) NOT (mice OR mouse OR rat OR rats))]) limited by timespan = 1985-2021; language = English; document type = articles |
| Scopus  （n=614） | (TITLE-ABS-KEY (((((asymptomatic) OR (subclinical)) OR (preserved AND ejection AND fraction)) OR (preserved)) OR (ejection AND fraction)) AND TITLE-ABS-KEY (valve) AND TITLE-ABS-KEY(((((stenosis) OR (aortic AND valve AND stenosis)) OR (aortic AND stenosis)) OR (aortic))) AND TITLE-ABS-KEY(((((global AND longitudinal AND strain) OR (gls)) OR (longitudinal AND strain)) OR (strain)) OR (speckle AND tracking)) AND TITLE-ABS-KEY ((((echocardiography) OR (echocardiography)) OR (echocardiogr*)) OR (ultrasound)) AND NOT TITLE-ABS-KEY (((case AND reports) OR (comment)) OR (editorial))) |

**Supplement Table 1. The full search strategy**
