# Supplementary material for: Prognostic Value of Global Longitudinal Strain in Asymptomatic Aortic Stenosis: A Systematic Review and Meta-Analysis
Source: Front Cardiovasc Med. 2022 Feb 18;9:778027. doi: 10.3389/fcvm.2022.778027 (PMC8894446; doi:10.3389/fcvm.2022.778027)
Supplement: Supplementary Table 2 — Quality assessment using Newcastle Ottawa scale. [file Table_2.docx]

**Supplement Table 2. Quality assessment using Newcastle Ottawa scale**

| **First Author, Year**  **(Ref. #)** | **Representativeness of exposed cohort** | **Selection of non exposed cohort** | **Ascertainment of exposure** | **Outcome not present at start of study** | **Comparability** | **Assessment of outcome** | **Follow-up long enough for outcomes to occur** | **Adequacy of follow up** | **Quality score** |
| --- | --- | --- | --- | --- | --- | --- | --- | --- | --- |
| Thellier et al., 2020 [13] | 1 | 1 | 1 | 1 | 2 | 1 | 1 | 1 | 9 |
| **First Author, Year**  **(Ref. #)** | **Case definition** | **representativeness** | **Selection of controls** | **Definition of controls** | **Comparability** | **Ascertainment of exposure** | **Same method of ascertainment** | **Non-response rate** | **Quality score** |
| Kitano et al., 2020 [12] | 1 | 1 | 0 | 1 | 1 | 1 | 1 | 1 | 7 |
| Gu et al., 2018 [14] | 1 | 1 | 0 | 1 | 1 | 1 | 1 | 1 | 7 |
| Carstensen et al., 2015 [15] | 1 | 1 | 0 | 1 | 1 | 1 | 1 | 1 | 7 |
| Nagata et al., 2014 [16] | 1 | 1 | 0 | 1 | 2 | 1 | 1 | 1 | 8 |
| Yingchoncharogen et al., 2012 [17] | 1 | 0 | 0 | 1 | 1 | 1 | 1 | 1 | 6 |
| Kearney et al., 2012 [18] | 1 | 1 | 0 | 1 | 2 | 1 | 1 | 1 | 8 |
| Zito et al.,2011 [19] | 1 | 0 | 0 | 1 | 1 | 1 | 1 | 1 | 6 |
| Lancellotti et al., 2010 [20] | 1 | 1 | 0 | 1 | 2 | 1 | 1 | 1 | 8 |
