# Supplementary material for: Prognostic Value of Global Longitudinal Strain in Asymptomatic Aortic Stenosis: A Systematic Review and Meta-Analysis
Source: Front Cardiovasc Med. 2022 Feb 18;9:778027. doi: 10.3389/fcvm.2022.778027 (PMC8894446; doi:10.3389/fcvm.2022.778027)
Supplement: Supplementary Table 3 — The outcomes of included studies. [file Table_3.docx]

| **First Author, Year (Ref. #)** | **Outcomes of included studies**  **Supplement Table 3. The outcomes of included studies** |
| --- | --- |
| Kitano et al., 2020 [12] | The primary end point was a composite of cardiac events, including cardiac death, heart failure requiring hospitalization, myocardial infarction or ventricular tachyarrhythmia. |
| Thellier et al., 2020 [13] | All-cause mortality |
| Gu et al., 2018 [14] | The primary outcome was a combination of aortic valve intervention (because of developing symptoms or EF <50%), hospitalization for heart failure and all-cause mortality. The secondary outcome was all-cause mortality. |
| Carstensen et al., 2015 [15] | The combined endpoint was indication for aortic valve replacement and sudden cardiac death. |
| Nagata et al., 2014 [16] | The primary endpoint was MACE including cardiac death, sustained ventricular tachyarrhythmia, aortic valve replacement, and hospital admission for heart failure within 2 years of follow-up. |
| Yingchoncharogen et al., 2012 [17] | The composite end point was cardiac death and aortic valve replacement necessitated by symptom development. Information regarding cause of death was obtained from review of death certificates. Only cardiac death directly related to AS (death from congestive heart failure or sudden death) was included as an outcome. |
| Kearney et al., 2012 [18] | MACE was defined as death or hospitalization due to cardiac causes and included cardiac mortality, non-fatal myocardial infarction, congestive cardiac failure, arrhythmia, cerebrovascular accident, aortic valve replacement (surgical and percutaneous) and other AS-related admission such as syncope, aortic valvuloplasty or angina. |
| Zito et al.,2011 [19] | The combined end point of the study included the development of significant symptoms (angina, dyspnea, syncope), cardiac death, and the clinical need for aortic valve replacement. |
| Lancellotti et al., 2010 [20] | Predefined endpoints for assessing the outcome were the occurrence during follow-up of symptoms, aortic valve replacement or death. |

AS = aortic valve stenosis; EF = ejection fraction.
