# Supplementary material for: Prognostic Value of Global Longitudinal Strain in Asymptomatic Aortic Stenosis: A Systematic Review and Meta-Analysis
Source: Front Cardiovasc Med. 2022 Feb 18;9:778027. doi: 10.3389/fcvm.2022.778027 (PMC8894446; doi:10.3389/fcvm.2022.778027)
Supplement: Supplementary Table 4 — Meta-regression of baseline feature on major adverse cardiovascular events (MACE) at follow-up. [file Table_4.docx]

| **Covariate** | **Coefficient** | **LCI** | **UCI** | **P Value** |
| --- | --- | --- | --- | --- |
| Male, % | 0.007 | -0.019 | 0.034 | 0.600 |
| Mean age, year | 0.056 | -0.022 | 0.133 | 0.160 |
| Mean BMI, kg/m^2^ | 0.022 | -0.222 | 0.265 | 0.862 |
| Mean BSA, m^2^ | 0.534 | -3.824 | 4.890 | 0.810 |
| Hypertension, % | 0.001 | -0.012 | 0.013 | 0.918 |
| DM, % | 0.012 | -0.019 | 0.042 | 0.447 |
| Dyslipidemia, % | -0.010 | -0.039 | 0.018 | 0.473 |
| CAD, % | -0.001 | -0.018 | 0.016 | 0.890 |
| CKD, % | -0.001 | -0.029 | 0.026 | 0.924 |
| Follow up, months | 0.016 | -0.022 | 0.054 | 0.404 |
| Mean AVA, cm^2^ | -0.442 | -2.444 | 1.560 | 0.665 |
| Mean iAVA, cm^2^/m^2^ | -1.344 | -2.990 | 0.301 | 0.109 |
| Mean AV Vmax, m/s | -0.036 | -1.354 | 1.281 | 0.957 |
| SVI, ml/m^2^ | 0.055 | -0.259 | 0.369 | 0.732 |
| LVMI, g/m^2^ | 0.017 | -0.036 | 0.071 | 0.525 |
| E/A | -0.901 | -2.557 | 0.756 | 0.287 |
| E/e’ | 0.002 | -0.080 | 0.084 | 0.960 |
| GLS threshold | 0.175 | -0.512 | 0.862 | 0.618 |
| Mean PG, mmHg | 0.000 | -0.017 | 0.017 | 0.976 |
| Mean EF, % | 0.039 | -0.175 | 0.253 | 0.721 |
| Mean GLS, % | -0.108 | -0.384 | 0.168 | 0.444 |

**Supplement Table 4. Meta-regression of baseline feature on MACE at follow-up**

AVA = aortic valve area; AV Vmax = Peak velocity of the aortic valve; CAD = coronary artery disease; DM = diabetes mellitus; EF = ejection fraction; GLS = global longitudinal strain; iAVA = indexed aortic valve area; LCI = low confidence interval; LVEF = left ventricle ejection fraction; LV-GLS = left ventricular global longitudinal strain; LVMI = left ventricular mass index; PG = pressure gradient; SVI = stroke volume index; UCI = upper confidence interval.
