# Supplementary figures and images for: Prognostic Value of Global Longitudinal Strain in Asymptomatic Aortic Stenosis: A Systematic Review and Meta-Analysis
Source: Front Cardiovasc Med. 2022 Feb 18;9:778027. doi: 10.3389/fcvm.2022.778027 (PMC8894446; doi:10.3389/fcvm.2022.778027)

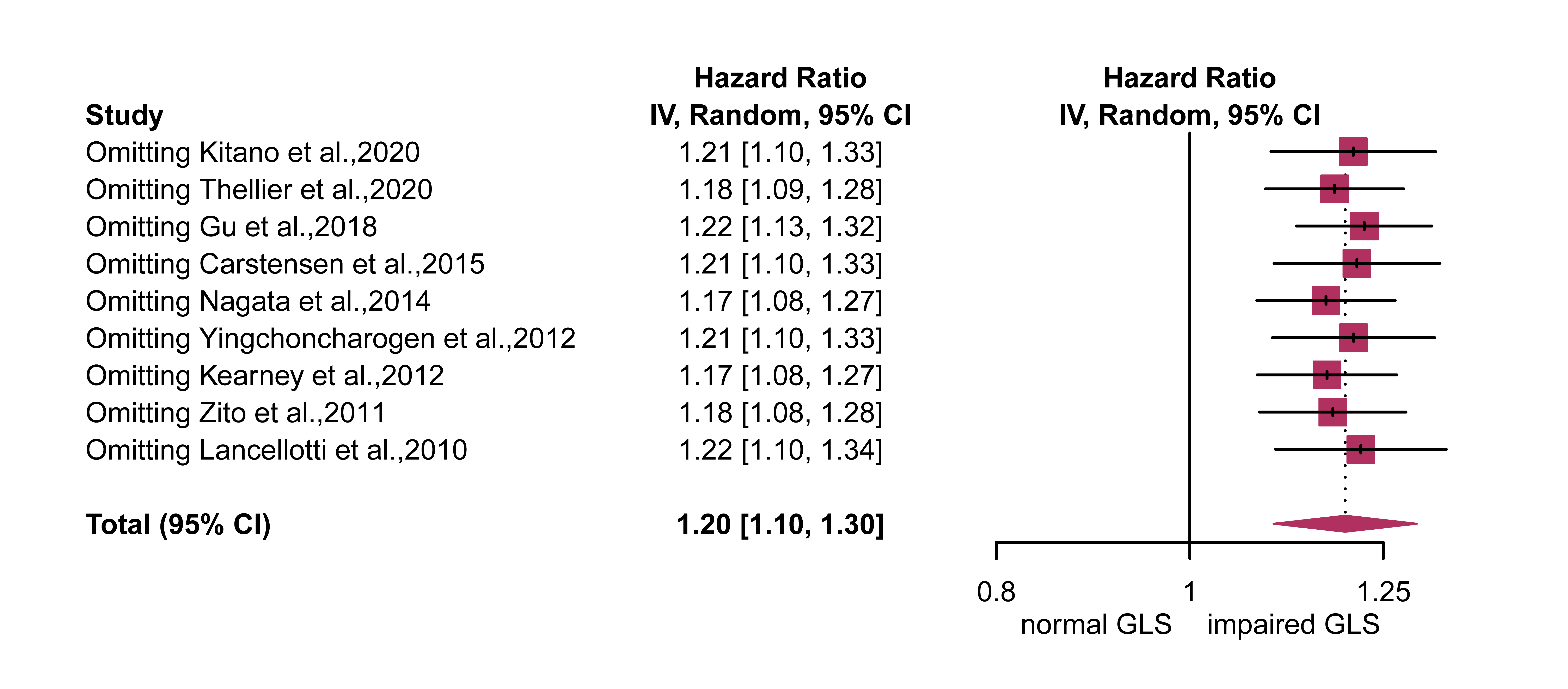

Supplement: Supplementary Figure 1 — Sensitivity analyses using the leave-one-out approach. [file Image_1.TIF]

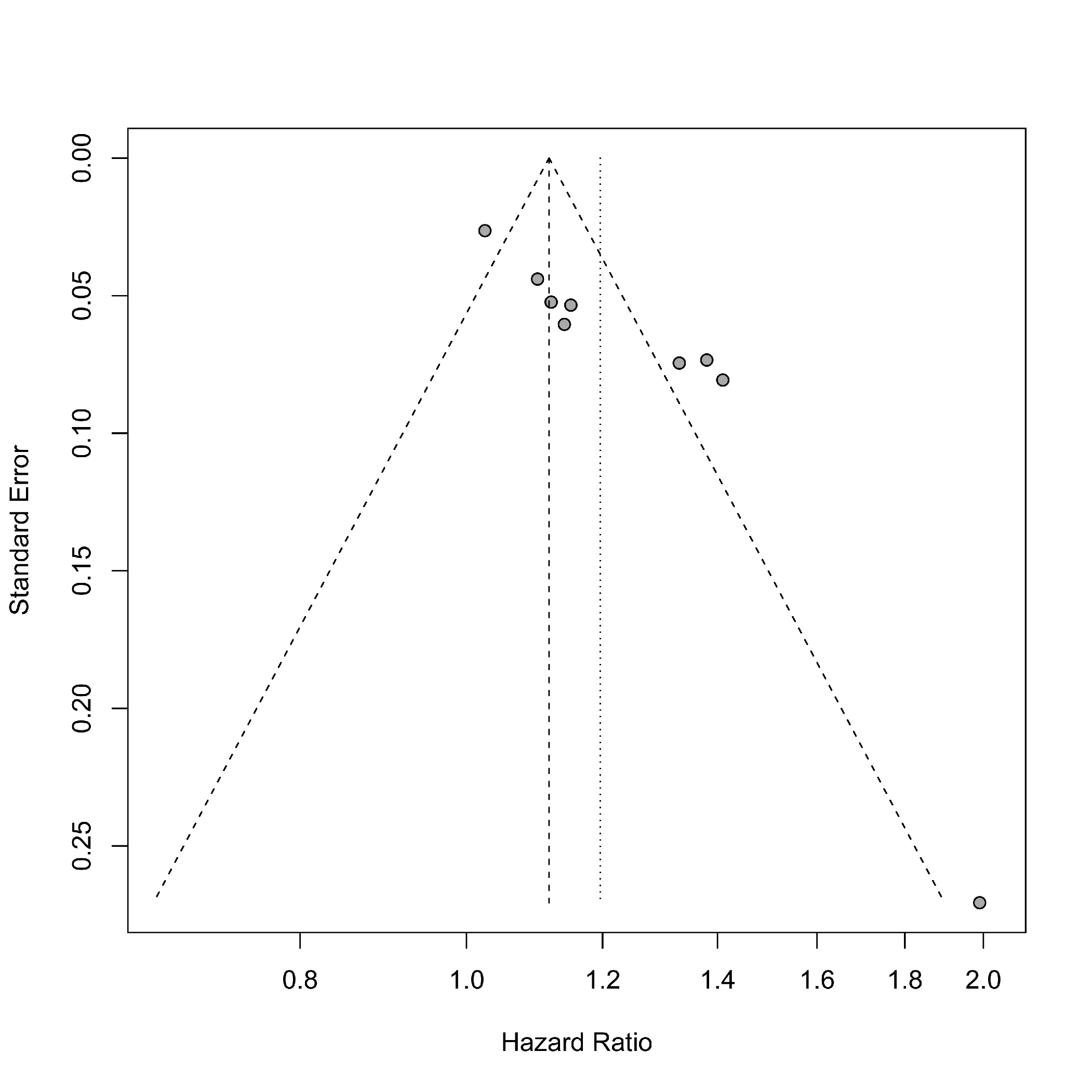

Supplement: Supplementary Figure 2 — Funnel plot for the association between global longitudinal strain (GLS) and major adverse cardiovascular events (MACE) in asymptomatic patients with aortic stenosis (AS). [file Image_2.TIF]
